# Micro-CT study of the root canal anatomy of maxillary canines

**DOI:** 10.4317/jced.54235

**Published:** 2017-10-01

**Authors:** Hugo Plascencia, Álvaro Cruz, Claudia-Azucena Palafox-Sánchez, Mariana Díaz, Claudia López, Clovis-Monteiro Bramante, Bertram I. Moldauer, Ronald Ordinola-Zapata

**Affiliations:** 1 DDS, Endodontic Postgraduate Program, CUCS-CUAltos, University of Guadalajara, Mexico; 2 DDS, MSc, PhD, Endodontic Postgraduate Program and Research Institute in Biomedical Sciences, CUCS, University of Guadalajara, Mexico; 3 DDS, MSc, PhD, Research Institute in Biomedical Sciences, Department of Medical Clinics, CUCS, University of Guadalajara, Mexico; 4 Postgraduate Student, Endodontic Postgraduate Program, CUAltos, University of Guadalajara, Mexico; 5 DDS, MSc, PhD, Department of Endodontics, Dental School of Bauru, University of Sao Paulo, Bauru, Brazil; 6 DDS, MS, Adjunct Assistant Professor in Endodontics and Surgical Course Co-Director, Nova Southeastern University, College of Dental Medicine, Fort Lauderdale, Florida

## Abstract

**Background:**

This study aimed to describe the anatomy of maxillary canines from a Western Mexican sub-population using micro-computed tomography (micro-CT).

**Material and Methods:**

Maxillary canines (n=32) were scanned at 19.6µm voxel resolution. Number and location of canals, the distance between the cemento-enamel junction and apex, occurrence of accessory and lateral canals, presence of oval canals, number of foraminas as well as two- (area, perimeter, roundness, aspect ratio, major and minor diameters) and three-dimensional (volume, surface area, and SMI) analysis were performed. Data of two-dimensional analyses at 5 different apical levels was statistically compared using Kruskal-Wallis tests (α=0.05).

**Results:**

Overall, 31 specimens had one root with a main canal (Vertucci type I). Mean distance from the apex to the cemento-enamel junction was 16.32±2.27. Apical foraminas were present in 14 specimens (43.75%). No statistical differences were found in the two-dimensional analyses between the foramen and the 1 and 2mm apical levels (*P* >0.05).

**Conclusions:**

Maxillary canines presenting one root canal were present in a high percentage of cases (96%). The prevalence of long oval canals was <12% at the apical third and at least 37% of the sample showed more than one point of exit in the last apical 3mm.

** Key words:**Maxillary canine, micro-computed tomography, root canal anatomy.

## Introduction

In the oral cavity, maxillary and mandibular canine plays an important role from the esthetic and functional point of views. Maxillary canines present a single root and in the majority of cases a single root canal (1,2). From the endodontic aspect, a great emphasis has been given to the transversal anatomy of this tooth, which in comparison to the central and lateral maxillary incisors presents an ovoid pulp chamber and a root canal that is wider labio-lingually than mesio-distally (1).

The apical region of maxillary canines has been studied in several in vitro experiments by using different methods as histology (3), radiographs (4-6), microscopic inspection of cross-sections of the tooth (2,7-14), clearing (15-22), scanning electron microscope (23) or cone beam computed tomography (24). Despite the relevant information that has been gained using these techniques, several limitations are also inherent to these studies, such as the lack of morphometric and tridimensional analysis of the same specimens. Micro-computed tomography (micro-CT) has been used in several studies aiming to obtain an accurate description of several parameters of the apical third with endodontic interest being apical diameters, roundness or volume (25-27). In spite of this method having been applied to several anatomies as mandibular incisors, canines, premolars and molars, to date no data can be found in the literature addressing the morphometric parameters of maxillary canines. Thus, the aim of this study is to analyze in vitro several morphometric parameters of maxillary canines from a specific, identified sub-population.

## Material and Methods

After ethics committee approval a total of 32 maxillary canines from a Western Mexican sub-population that were extracted due to non-restorable caries or periodontal diseases without internal or external resorptions were selected. The patient gender and age were unknown. The methods of micro-CT acquisition followed the guidelines of previous studies (25-27), which included a custom attachment to fix the teeth in a vertical position and a micro-computed tomographic system SkyScan 1174 (Bruker-microCT, Kontich, Belgium) calibrated with the following parameters: 50kV, 800mA, and an isotropic voxel size resolution of 19.6μm. The radiographic images of each tooth were reconstructed to the BMP format using the NRecon v.1.6.9.8 software (Bruker-microCT), as a result between 700-1000 axial cross-sections of the roots were available. Three-dimensional models were reconstructed using the P3G format from the transversal sections after automatic segmentation and binarization process by using the CTAn v.1.14.4.1 software (Bruker-microCT). CTVol v.2.2.3.0 software (Bruker-microCT) was used for visualization and qualitative evaluation of the specimens.

DataViewer v.1.5.0.2 software (Bruker-microCT) was used to evaluate the number and location of root canals, the presence of accessory and lateral canals, position of foramina’s or points of exit at the apical third, the distance between the apex and the last apical foramina and the distances between apex and the cemento-enamel junction. The CTAn v.1.14.4.1 software (Bruker-microCT) was used for the two-dimensional (area, perimeter, roundness, aspect ratio, major diameter and minor diameter) at the foramen and from the 1 to 5mm apical levels. Three-dimensional evaluations of the root canal system included the analysis of volume, surface area, and structure model index.

Two-dimensional evaluations follow the ASTM definitions and have been previously described (25-27). Area and perimeter were calculated using the Pratt algorithm. The cross-sectional appearance, round or more ribbon-shaped, was expressed as roundness. Roundness of a discreet two-dimensional object is defined as 4.A/(π.(dmax)2), where “A” is the area and “dmax” is the major diameter. The value of roundness ranges from 0 to 1, with 1 meaning the perfect circle. The major diameter was defined as the distance between the two most distant pixels in that object. The minor diameter was defined as longest chord through the object that can be drawn in the direction orthogonal to that of the major diameter. Volume was calculated as the volume of binarized objects within the volume of interest (VOI). For the measurement of the surface area of the three-dimensional multilayer dataset, two components to surface measured in two dimensions were used; first the perimeters of the binarized objects on each cross-sectional level, and second, the vertical surfaces exposed by pixel differences between adjacent cross-sections (25). Structure model index (SMI) involves a measurement of surface convexity in a three-dimensional structure (28). An ideal plate, cylinder and sphere have SMI values of 0, 3 and 4, respectively. The prevalence of long oval canals was determined according to Wu *et al.* (2), that is when the aspect ratio was ≥2.

The results of two and three-dimensional analysis, as well as, the distances between the anatomical landmarks were described as mean, standard deviation and range. Because normality assumptions could not be verified (Shapiro-Wilk test; *P*>0.05), data from the two-dimensional analysis at every apical level were statistically compared by using Kruskal-Wallis Dunn test and described using median and range values. The statistical analysis was performed using SPSS v20.0 for Windows (SPSS Inc., Chicago, IL) with a significance level set at 5%.

## Results

-Qualitative analysis

The analysis of the internal anatomy of maxillary canines showed that all canals presented a Vertucci’s type I anatomy, except for one tooth that presented a type III and one a non-Vertucci anatomy. The mean distance and standard deviation in mm between the cement-enamel junction until the apex was 16.32±2.27, with a range of 11.78-21.9 mm.

In 14 teeth (43.75%) were observed accessory canals in the last apical 5mm, totaling 51 branches. In these cases, the mean distance between the apex and the last apical foramina was 2.5mm with a range of 0.82 to 4.97mm. Most of the foramina’s were present within the 3 mm apical level (n=12, 37%) and 2 foraminas were present between the 3 and 5mm apical level. Also, 3 lateral canals were found between 6-12mm from the apex. The results regarding the localization of the main foramen showed that only one case presented a centralized position (3.12%) and deviated from the anatomic apex in 31 cases (96.87%). The most frequent locations of deviation were toward the buccal side in 22 cases (70.96%), followed by the palatal side (7 cases) and mesial side (2 cases).

-Quantitative analysis

**Figure 1:**
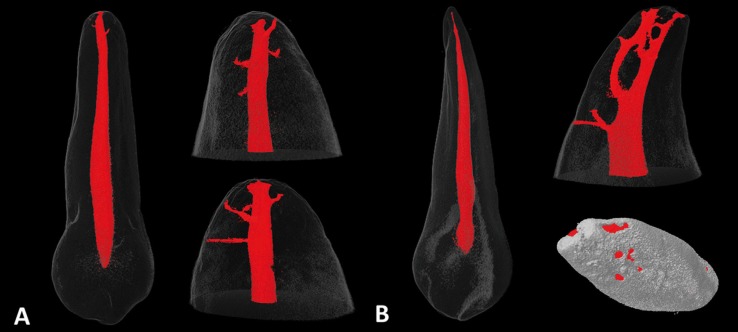
Representative micro-CT tridimensional reconstructions showing a Vertucci type I anatomy (A) and a non-classified anatomy, that includes the presence of a lingual canal at the apical third (B). The presence of numerous ramifications can be also observed in both cases.

Overall, mean volume, surface area, and SMI of the root canal system were 12.44±6.60mm3, 81.35±32.50mm2, and 2.96±2.18, respectively. Data from two-dimensional analysis of the root canals at the 5 apical mm are presented in  Table 1. No statistical difference was found in the comparison of area, perimeter, roundness, aspect ratio, major and minor diameters values among the foramen and the 1-2 apical mm levels (*P*>0.05). Other statistical significances are shown in Table 1. Long oval canals (aspect ratio >2) were present in 3, 3, 2, 3 and 4 cases between the 1 and 5mm apical level (9.3-12.5%). See  Table 2. Representative ima-ges of the studied anatomies are found in Figure 1.

**Table 1:**
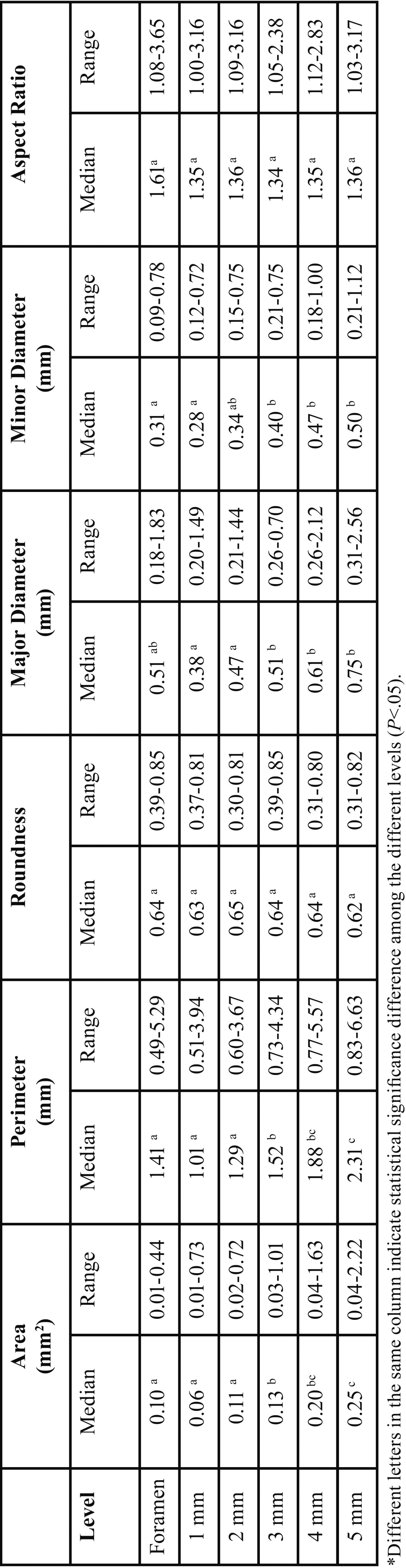
Morphometric two-dimensional analysis at the apical level of maxillary canines (n=32).

**Table 2:**
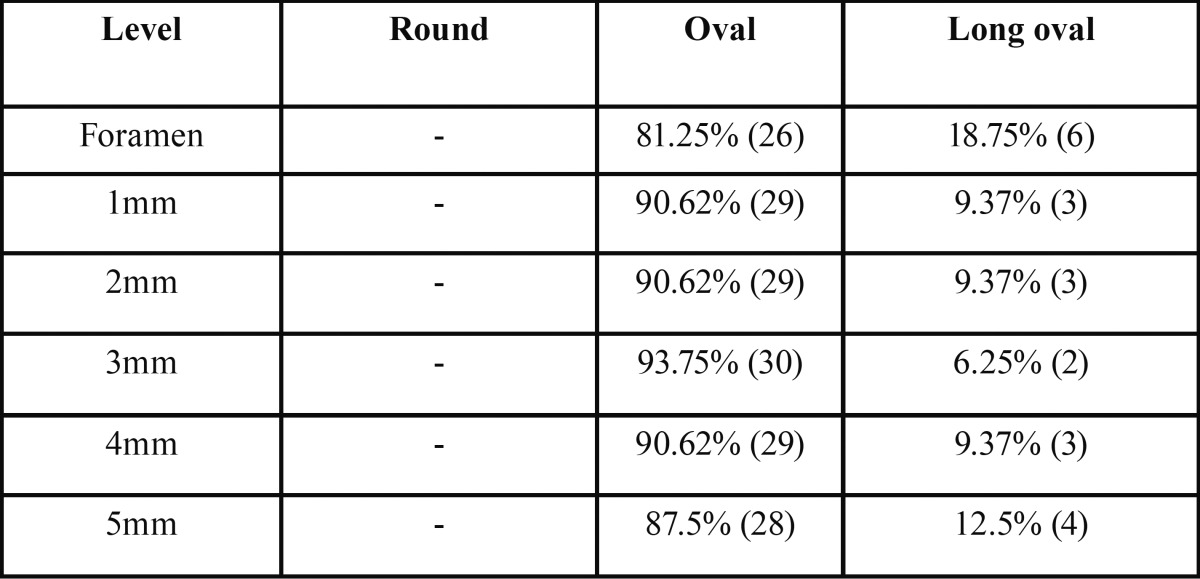
Percentage frequency (n) of canal shapes observed in the apical region of the maxillary canine (n=32). A long oval canals was determined is when the aspect ratio was ≥2. An oval canal presents an aspect ratio higher than 1 but less than 2.

## Discussion

There is an agreement among several studies that the more prevalent Vertucci configuration in maxillary canines is the type I in the 91-100% of the cases (17,19,29), results that are confirmed in the present study. However, other authors have found different results regarding the presence of a single canal as 75% (20) or 81% (24) mainly in Asian populations.  Table 3 summarizes the main results of these studies. Most common used methods to study the anatomy of maxillary canines were the clearing technique and cross-sections (2,10,16,17), one study used scanning electron microscope (23) and other used the CBCT technique (24). Despite the considerable data about the number of root canals of maxillary canines, a literature review ( Table 3,  Table 3 continue) shows that there is a lack of information describing several morphometric parameters with endodontic interest using current digital technology.

**Table 3:**
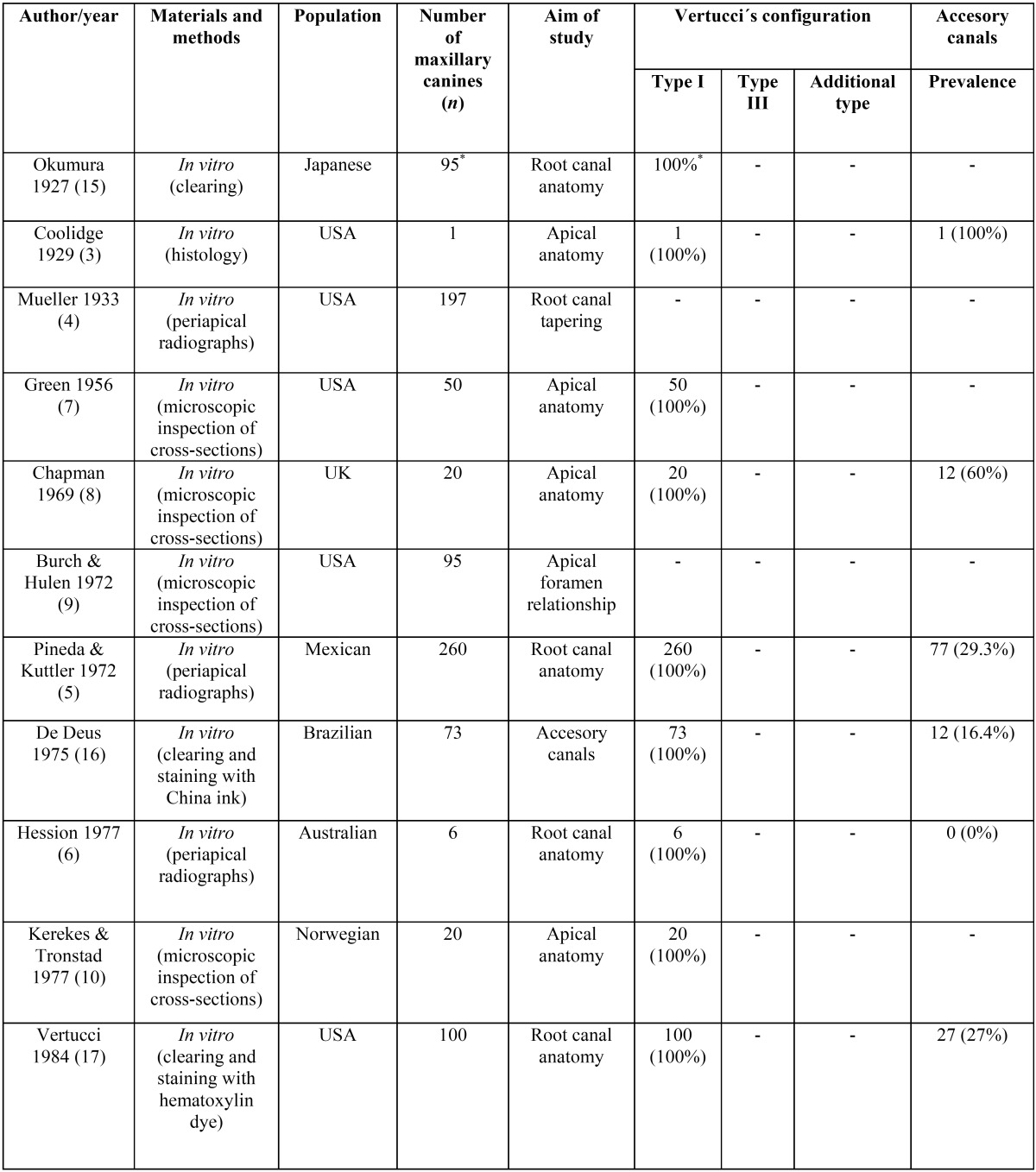
Maxillary canine: number of root canals and prevalence of accessory canals (23 articles).

**Table 3 continue:**
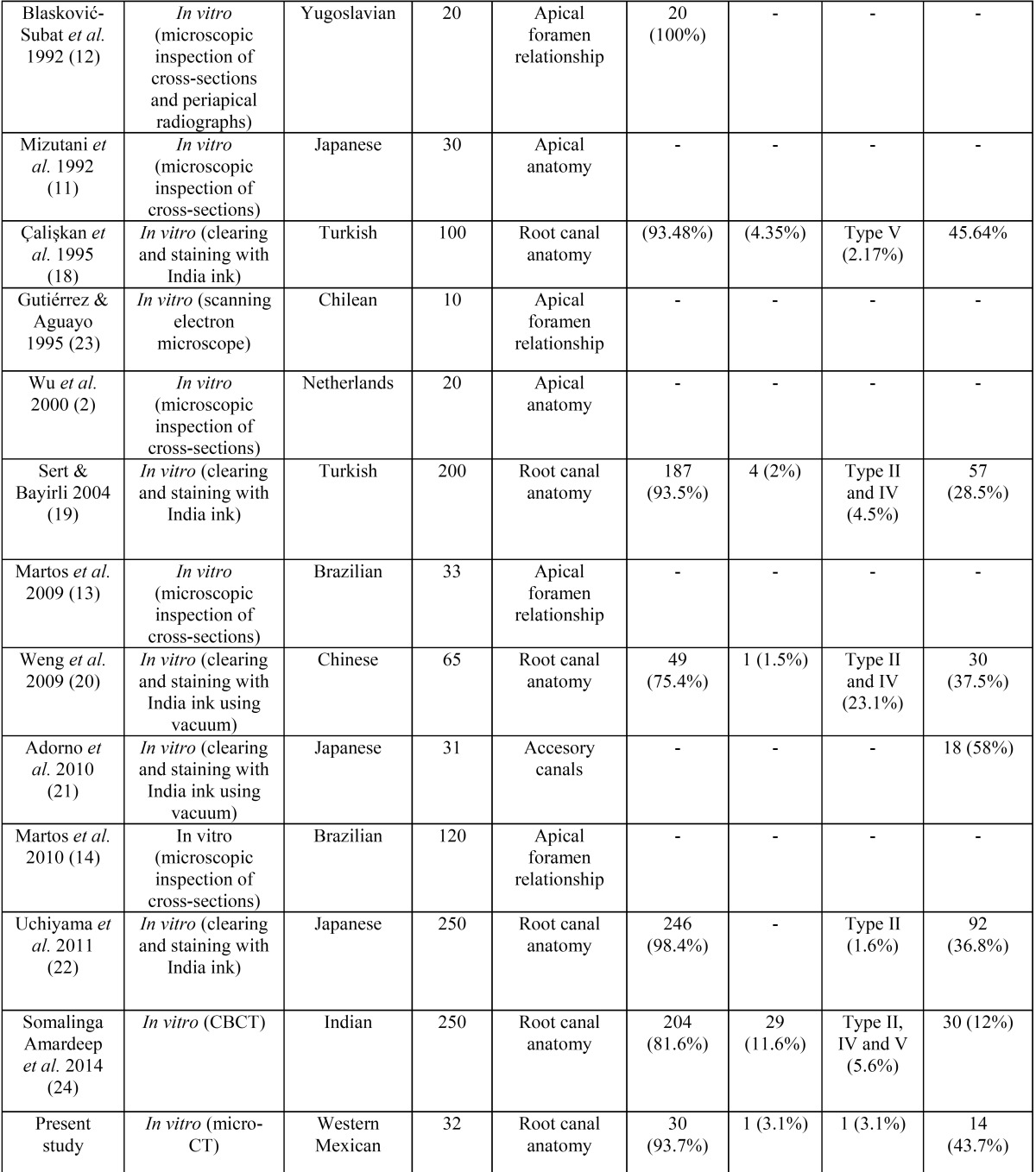
Maxillary canine: number of root canals and prevalence of accessory canals (23 articles).

At least two studies addressed the apical diameter of maxillary canines at the apical third (2,10). However, no studies using morphometric micro-CT analysis were found in the literature. The bidimensional analysis showed that the last apical mm present in overall a major diameter of 380.77μm, which approximately corresponds to the diameter of a 0.40mm ISO file. From this point, the taper of the apical third increases in a coronal direction until a diameter of 750.16μm (approx. 0.80mm ISO file) at the 5mm level. Similar results were reported in the study of Kerekes & Tronstad (10), who determined at least a diameter of 0.45mm to obtain a circular shape at the 1mm apical level. Wu *et al.* (2) found lower apical diameters values as 0.31mm with a range of 0.16-0.58mm. One interesting point is that the apical diameter, area and perimeter of the last apical mm in maxillary canines present in overall lower values in comparison to the mandibular canine, where the mean of apical diameter is 0.59mm as reported in a previous micro-CT based study (30).

Another topic reported in the endodontic anatomy literature is the tridimensional analysis of the root canal system. Limited reports regarding this topic can be found in maxillary anterior teeth. In a previous work (30), several morphometric aspects of mandibular canines were analyzed. The results show that in terms of pulpal space volume and SMI, both maxillary and mandibular teeth present similarities. Volume values were 12.44mm3 and 13.33mm3, and SMI 2.96 and 3.35 for maxillary and mandibular canines respectively.

The position and number of apical foramina’s is another topic continuously described in the endodontic literature with relevance for apical surgery. At least 13 studies investigated the position of the main apical foramen in maxillary canines (5,7-9,11-14,17-19,22,24). The most common finding of these studies (8,9,12-14) is that the apical foramen was located in an eccentric position towards the buccal side similar to the results found in this study (96%). However, Uchiyama *et al.* (22) reported that eccentricity was found in only 30.5% of the cases in a Japanese sub-population. Regarding the number of apical foraminas different results can be found in the literature. The prevalence can vary from 15% (7,8) to 70% (23). In the present study, 43% of the sample presents more than 1 apical foramen, with a range between 2 and 9 in the last apical 5mm. The prevalence of accessory canals was higher than the study of De-Deus (16) and Adorno *et al.* (21), that found a prevalence of 15% and 19%. One important point is that at least 12 of the 14 roots that presented more than one apical foramen showed that the last apical foramina was located between the 3mm apical level (37.5%), a value that is commonly considered for apical resection procedures in periapical surgery.

Regarding the prevalence of long oval canals, Wu *et al.* (2) reported that the presence of these canals in which the major canal diameter was at least two times the short diameter, was between 0-6% in the last 5 apical mm of maxillary canines. This is similar with our findings where the canals were long oval at 1 mm in 9.3% (3 cases) of 32 inspected maxillary canines. At the other apical levels, the prevalence was between 9%-12.5%. Statistical analysis of roundness and aspect ratio values, showed that a similar cross-sectional geometry could be found in the last 5 apical mm of maxillary canines. Therefore, we can conclude that based on two-dimensional morphometric findings, there is a low tendency of canine root canals to be long oval regardless of the apical region analyzed. The roundness values reported in this study are similar to the values found in mandibular canines (0.68) (30), but higher to the values of mandibular incisors, where the roundness values tend to be lower as 0.4-0.5 including an increased prevalence of long oval canals in the last apical millimeters (76.2%), specially in the Vertucci type 3 variation (26).

In conclusion, maxillary canines presenting one root canal were detected in a high percentage of cases (96%). The prevalence of long oval canals was <12% at the apical third and at least 43% of this anatomy presents multiple accessory canals and foraminas.
